# Verbal behavior in Alzheimer’s disease patients: Analysis of phrase repetition

**DOI:** 10.1590/S1980-57642010DN40300008

**Published:** 2010

**Authors:** Juliana Francisca Cecato, José Eduardo Martinellil, Luana Luz Bartholomeu, Ana Paula Basqueira, Mônica Sanches Yassuda, Ivan Aprahamian

**Affiliations:** 1 Instituto de Geriatria e Gerontologia de Jundiaí e Faculdade Anhanguera de Jundiaí, Jundiaí SP, Brazil.; 2 Instituto de Geriatria e Gerontologia de Jundiaí e Faculdade de Medicina de Jundiaí, Jundiaí SP, Brazil.; 3 Faculdade Anhanguera de Jundiaí.; 4 Faculdade Anhanguera de Jundiaí, Jundiaí SP, Brazil.; 5 EACH, Universidade de São Paulo, São Paulo SP, Brazil.; 6 IPq HCFMUSP e Hospital Albert Einstein, São Paulo SP, Brazil.

**Keywords:** verbal behavior, elderly, Alzheimer’s disease, language, cognitive tests

## Abstract

Language problems in the elderly with AD are due to the fact that deterioration occurs not only in semantic memory, but in a group of cognitive factors, evidenced by a deficiency in search strategies for linguistic information.

**Objectives:**

To evaluate phrase repetition in two cognitive tests, the MMSE and MoCA, in a group of Alzheimer disease patients (AD) and normal controls.

**Methods:**

A Cross-sectional study was conducted involving 20 patients who sought medical assistance at a geriatric institute in Jundiaí, São Paulo. The subjects underwent a detailed clinical examination and neuropsychometric evaluation. All subjects with AD met DSM-IV and NINCDS-ADRDA criteria. Ten patients received a diagnosis of AD and 10 were healthy subjects, forming the control group (CG).

**Results:**

All participants correctly answered the phrase from the MMSE (phrase 1). The MoCA phrases (phrases 2 and 3) were correct in 80% and 90%, respectively in the CG and in 40% and 50%, respectively in the AD group.

**Conclusions:**

The MoCA test proved more effective in evaluating the echoic behavior in AD patients compared to the MMSE. The simpler phrase repetition task in the MMSE was found to be less sensitive in detecting mild language decline in AD patients.

## Abstract (pt)

As dificuldades com a linguagem em idosos com Alzheimer (DA) se devem ao fato de que a deterioração não está relacionada apenas à memória semântica, mas um conjunto de fatores cognitivos, os quais demonstram a deficiência nas estratégias de busca de informações lingüísticas.

**Objetivos:**

Avaliar a repetição de frases em dois testes cognitivos (MEEM e MoCA), em um grupo de participantes normais e outro com o diagnóstico de DA.

**Métodos:**

Estudo transversal com 20 pacientes que procuraram o serviço de saúdo no instituto de geriatria em Jundiaí, São Paulo. Os participantes passaram por anamnese clínica detalhada e avaliação neuropsicométrica. O grupo de participantes com diagnóstico de DA foi feito pelos critérios do DSM-IV e NINCDS-ADRDA. Dez pacientes receberam o diagnóstico de DA e 10 como participantes normais, formando o grupo controle (GC).

**Resultados:**

Todos os participantes responderam corretamente a frase do MEEM. As frases do MoCA (frase 2 e 3) foram corretas em 80% e 90% respectivamente no GC. O grupo com DA respondeu corretamente 40% e 50% respectivamente.

**Conclusões:**

O teste MoCA mostrou ser mais eficiente em avaliar o comportamento verbal ecóico em pacientes com DA quando comparados ao MEEM. A simples repetição de frase no MEEM mostrou ser menos sensível para detectar declínio leve de linguagem em pacientes com DA.

Verbal behavior is a complex operating behavior which is maintained by listener’s feedback in such a way that both subjects have similar verbal repertoire, trained naturally by the environment.^1-4^ It has special operant characteristics, as it has an indirect and non-mechanical influence with the physical environment. Nevertheless, its effects directly change human behavior through the relationship with other subjects.^1-4^

In the elderly, verbal behavior and the linguistic abilities receive greater attention due to their sensitivity in the diagnostic investigation of dementia.^5,6^ Language disorders emerge frequently in dementias.^7^ The incidence of language difficulties in dementia syndromes ranges from 88 to 95% and in the case of Alzheimer’ disease (AD), these communication disturbances may afflict 100% of patients.^5^

Verbal behavior deficits in the elderly are frequently interpretated as symptoms of dementia.^7^ Along these lines, Amieva et al.^8^ found that healthy subjects who evolved to AD presented language decline on objective verbal fluency tasks twelve years before the diagnosis.

Syntactic complexity seems to be reduced in patients with dementia compared with elderly healthy subjects as is formulation of short phrases, while impaired syntactic comprehension are also characterized.^9^ Some language functions remain preserved in the earlier stages of dementia such as comprehension, syntax and repetition of phrases.^7,10^ The language problems in the elderly with AD are due to the fact that deterioration occurs not only in semantic memory, but in a group of cognitive factors. This deficiency is evidenced by the search strategies used for linguistic information.^7,10^ In a 1987 study, the Alzheimer group score was worse in phrase repetition compared with healthy elderly subjects. Nevertheless, both groups (normal controls and AD group) had the same score on other cognitive tests.^11^

In a review, Benke et al.^7^ described that cognitive evaluations may not be effective for understanding how language deficits work in dementia and aphasias. In the elderly, the neuropsychological tests should evaluate several domains including speech, naming, repetition and several others, to reach an effective differential diagnosis in cases of aphasia and dementia.^7,10,12^

The aim of this paper was to evaluate the echoic verbal behavior in the phrase repetition test from two cognitive instruments, the Mini-Mental State Examination (MMSE)^13^ and Montreal Cognitive Assessment (MoCA),^14^ in a group of AD patients and normal controls.

## Methods

A cross-sectional study involving 20 patients who sought medical assistance at a geriatric institute in Jundiaí, São Paulo was conducted between January 2009 and January 2010. The subjects underwent a detailed clinical examination and neuropsychometric evaluation. The tasks used were: the CAMDEX and its cognitive battery (CAMCOG),^15^ MMSE,^13^ verbal fluency with animals, fruits and words with the letter “M” versions,^6,16^ Clock Drawing test,^17^ Geriatric Depression Scale (GDS)^18^ and the MoCA test - Brazilian version.^14^ All tasks were applied as part of the routine local neuropsychological evaluation of patients with complaints of cognitive decline. AD patients with severe cognitive decline (Clinical Dementia Rating = 3), major depression, plegia or paresis, important tremor, functional impairment in both hands, severe visual or auditory impairment, and patients who refused to complete any of the tests, were excluded. All subjects with AD met DSM-IV^19^ and NINCDS-ADRDA criteria for probable AD.^20^ Inclusion criteria for the control group were neuropsychiatric tests scores above the cut-off points, without dementia criteria on DSM-IV, a GDS score lower than seven points and absence of functional impairments in daily living activities as informed by a relative or caregiver. The subjects were matched for education level. None of the patients were in use of any drug that potentially depresses the central nervous system at the time of the neuropsychological evaluation.

Ten patients received diagnosis of AD and 10 as healthy subjects, forming the control group (CG). Subjects in both groups were aged 60 years or older and had more than 8 years of formal education. The evaluations were carried out after the first clinical examination and the patients did not take any medication. All information were analyzed by the SAS System for Windows (Statistical Analysis System) 6.12 version and SPSS 15.0 (2007). To describe the profile of the sample, frequencies of categorical variables (gender and education) and descriptive statistics of continuous variables (age, scores from CAMCOG, MMSE and MoCA), the mean values, standard deviation, minimum, maximum and median were presented. Spearman’s coeficient was used to correlate the tests with the phrases from the MMSE (phrase 1) and from the MoCA (phrase 2 and 3). The independent samples test (T-test) was used to evaluate means and the significant differences among the tests. The phrases were: phrase 1=“Nem aqui, nem ali, nem lá”; phrase 2=“Eu somente sei que é João quem será ajudado hoje”; phrase 3=“O gato sempre se esconde embaixo do sofá quando o cachorro está na sala”.

## Results

Of the 20 subjects, 13(65%) were women. In the AD group, the age range was 65 to 91 years old (mean=76.7 yrs, median=76.5 yrs, standard deviation [SD]=±7.45yrs) and 10 participants had high educational level (>8 years). Mean age in the CG was 68.8 years old, (SD±4.66yrs). All participants in the CG had high educational levels.

In AD group, 9 subjects did not achieve the cut-off on the MMSE (mean=23.9, median=24, SD±3.98) and none achieved this score on the MoCA (mean=19.7 median=21, SD±3.74). On the CAMCOG battery (mean=78.5, median= 81, SD=11.32), 60% scored above the cut-off. The CG had high scores on the MMSE, MoCA and the CAMCOG (Table 1).

**Table 1:** Scores of 20 subjects on cognitive tests.

Tests	Group	N	Mean	Min.	Max.	SD
MMSE	AD CG	10 10	23.9 29.5	15 29	28 30	3.98 0.53
CAMCOG	AD CG	10 10	78.5 100.2	60 93	95 107	11.32 4.59
MoCA	AD CG	10 10	19.7 27.9	12 26	23 30	3.74 1.45

AD: Alzheimer's disease group; CG: control group; N: number of subjects; Min: minimum; Max: maximum; SD: standard deviation.

Analyses of the T-test results discriminated the two groups: AD and CG. This analysis showed that the mean in the CG was higher than that in the AD group on the MoCA (p<0.001), MMSE (p<0.001), CAMCOG (p<0.001), verbal fluency test animals (p<0.001), fruits (p<0.001), letter “M” (p=0.011) and age (p<0.001)(Table 2).

**Table 2:** T-test analyses of cognitive evaluations, age and education in 20 subjects by CG and AD.

	Groups	N	Mean	SD	Mean Std.Error	Sig. (2-tailed)
MoCA score	AD CG	10 10	**19.90** ** 27.90**	3.872 1.449	1.224 0.458	**<0.001**
MMSE score	AD CG	10 10	**23.80** **29.50**	3.910 0.527	1.236 0.167	**<0.001**
CAMCOG	AD CG	10 10	**79.40** ** 100.20**	11.965 4.590	3.784 1.451	**<0.001**
Mendez	AD CG	10 10	18.30 19.50	2.406 0.707	0.761 0.224	0.148
Shulman	AD CG	10 10	4.20 4.70	0.789 0.483	0.249 0.153	0.105
Sunderland	AD CG	10 10	9.10 10.00	1.912 0.000	0.605 0.000	0.154
VF Animals	AD CG	10 10	**10.30** **18.70**	4.900 3.592	1.550 1.136	**<0.001**
VF Fruits	AD CG	10 10	**7.80** ** 16.40**	1.814 3.688	0.573 1.166	**<0.001**
VF "M"	AD CG	10 10	**8.70** **14.50**	4.990 4.062	1.578 1.285	**0.011**

AD: Alzheimer's disease; CG: control group; N: number of subjects; VF: verbal fluency.

Comparing the repetition of phrases task, all 20 subjects (AD and CG) repeated the sentence correctly on the MMSE (phrase 1). On the MoCA, 6 patients in the AD group failed to repeat the first phrase (phrase 2) and 5 failed to repeat the second sentence (phrase 3). Despite failing to repeat the MoCA phrases, these subjects successfully repeated the MMSE phrase (Table 3).

**Table 3:** Scores of both groups on repetition phrases of the MMSE and MoCA.

Subjects	Answer	Phrase 1 n (%)	Phrase 2 n (%)	Phrase 3 n (%)
AD	Correct Error	10 (100%) 0	4 (40%) 6 (60%)	5 (50%) 5 (50%)
CG	Correct Error	10 (100%) 0	8 (80%) 2 (20%)	9 (90%) 1 (10%)

n: numbers of subjects.

All participants from the CG correctly repeated the MMSE phrase (100%). The MoCA phrases (phrases 2 and 3) were correct in 80% and 90% (respectively) in the CG. The AD group underperformed on the MoCA showing worse scores compared to those of the CG (Table 3).

## Discussion

This study compared phrase repetition in the MMSE and MoCA tests. On the MMSE, both groups successfully performed the language task of phrase repetition, but differed significantly compared to the MoCA. These findings can be explained by the fact that some aspects of language, such as the repetition of simple phrases (MMSE phrase) appear to be preserved in early and moderate stages of dementia.^21^ However, our findings showed that the more complex the sentence, the greater difficulty dementia patients have repeating them. These outcomes corroborate those of Knibb’s^9^ studies that found linguistic impairment in dementia. In tasks which required understanding of the meaning of the phrase,^5^ as observed in MoCA, AD patients had a worse score compared to controls. Similar results were found by Murdoch^11^ and recently by Báez et al.^22^ who assessed the performance of AD patients on tasks involving the interpretation of word meaning. In their sample of 60 subjects, patients with AD were found to have considerable difficulty in semantics.

The MoCA test seems to be a more robust instrument compared to the MMSE because it involves more words in the phrase repetition test, little evidence of learning and a long delay before word recall,^23^ explaining the worse performance in verbal behavior on the repetition of phrases. The complex phrases should be used for differentiating cases of dementia^7^ and healthy elderly with high education, because performance on language tests such as repeating complex phrases appears to be impaired in cases of Alzheimer’s disease.

Another important concern is with regard to levels of education. Less educated subjects can be at a higher risk for AD.^24^ The participants in this study had high levels of schooling, which showed the importance of education in the preservation of cognitive functions objectively assessed in the CAMCOG evaluation.^25^ High scores on the CAMCOG were reported by Aprahamian et al.^26^ whose study found new cut-off points on the CAMCOG test according to education. In subjects with a high level of education (=9 years) the cut-off point should be 90 points. In comparison, the mean score found in our AD group was 78.5, i.e. well below the score proposed by Aprahamian et al. while our control group had a mean score of 100.2.

Level of education should be considered in psychological evaluations because it can generate false positives in patients with low educational levels and false negatives in highly educated individuals.^27^ Another concern is regarding the capacity to concentrate in dementia patients.

To conclude, the MoCA phrase repetition test proved more effective in evaluating the echoic behavior in AD patients compared to the MMSE phrase. The simpler phrase repetition task on the MMSE was found to be less sensitive in detecting mild language decline in AD patients.

These results suggest that verbal behavior is a complex skill in the evaluation of dementia and is subject to environmental influences such as education. Findings also suggest that future studies, involving a large number of participants, are needed to investigate subtle linguistic changes in AD patients.
